# Team Ownership and Philanthropy in Professional Sport: A Perspective on Organizational Generosity

**DOI:** 10.3389/fspor.2022.798919

**Published:** 2022-03-31

**Authors:** Kathy Babiak, Daniel Yang

**Affiliations:** School of Kinesiology, University of Michigan, Ann Arbor, MI, United States

**Keywords:** sport team owners, charitable giving, upper echelon theory, social status, corporate social responsibility (CSR)

## Abstract

Corporate philanthropy (CP) is a vehicle for businesses to create a social impact in communities where their operations are located. An overlooked aspect of this phenomenon is the role and function played by CP influencers within firms—particularly organizational principals/owners. Using an upper echelons perspective, this study explores the relationship between team ownership and the level of CP in the professional sport context. To this end, longitudinal data of philanthropic giving of 94 U.S. professional sport teams in the NBA, NFL, MLB, and NHL were collected. We also collected team owner characteristics such as individual/family ownership, age, tenure as team owner, other charitable work, educational background, and connection to community from a variety of publicly available sources. The findings revealed that team owner age, ownership tenure, and previous philanthropic involvement contributed to increased charitable giving in professional sport team corporate foundations. Theoretical and practical implications of these findings are discussed in the paper.

## Introduction

As one of the pillars of corporate social responsibility (CSR), corporate philanthropy (CP) is a vehicle for businesses to create a social impact in communities where their operations are located and to lever access to markets nationally and globally (Gautier and Pache, 2015). The practice has been defined as “...gift giving or monetary contributions made by firms to social or charitable causes, such as education, the arts, health care, environmental protection, or disaster relief” (Gao et al., 2017, p. 277). Scholars have begun to explore this emerging phenomenon to understand the benefits of companies acting charitably. Specifically, researchers have examined the marketing and branding benefits associated with CP (McAlister and Ferrell, 2002; Ricks, 2005), governance issues around CP (Wang and Coffey, 1992; Bartkus et al., 2002; Petrenko et al., 2016), and the insurance benefits of CP in the face of ethics scandals or transgressions (Williams and Barrett, 2000). While scholarship has helped to shed light on our understanding of the strategic value of CP, researchers are still seeking to fully understand how individuals such as business leaders might influence the scope and impact of their company's CP (Marquis and Lee, 2013).

Petrenko et al. (2016) and Gao et al. (2017) noted that while the relationship between certain organizational determinants of CP has been identified and investigated in the literature (i.e., the size of the firm, firm financial performance, slack resources, institutional pressures, industry context, and corporate governance), there has been little research carried out to understand the characteristics and impact of key organizational decision makers on CP (Finkelstein et al., 2009). More recent scholarship posits that top decision makers (such as senior executives or CEOs) have significant control over CP choices (Sánchez, 2000; Hambrick and Quigley, 2014; Petrenko et al., 2016; Gao et al., 2017). While CP is carried out at the organizational level, there may be significant individual level drivers, suggesting that decisions around CP might be “...affected by owners' or top managers' characteristics, values and perceptions” (Gao et al., 2017, p. 278) among other things. Thus, many of a firm's choices may reflect top leaders or owners' unique experiences, values, and individual traits. It may be particularly significant to explore these factors given that individuals who lead or own businesses have agency around their socially oriented actions and can express their philanthropic priorities, values, and motivations through the medium of their businesses (Buchholtz et al., 1999; Marquis and Lee, 2013; Li et al., 2015).

In the professional sport context, team owners garner significant attention and have influence in their respective communities. The decisions they make can impact numerous civic issues through their team's activities including outcomes regarding public subsidy expenditures (sport facilities/stadium finance) (Swindell and Rosentraub, 1998; Jones, 2001), securing government and community support for urban planning and design projects (Chanayil, 2002; Friedman and Mason, 2004; Mason et al., 2017; O'Reilly, 2019), and social/community impact (Babiak and Wolfe, 2009; Sheth and Babiak, 2010). Furthermore, decisions made by team owners are often critical to the team's performance in the front office and on the field (for example, in decision making around hiring senior executive leadership (Audas et al., 2002), drafting talent and player salaries (Rosner and Shropshire, 2011), pricing strategies (Hayduk, 2021), and strategic decision making (i.e., structure, rules and regulations, policies, etc.) for their respective leagues (Késenne, 2014). Thus, because professional sport businesses are privately held (vs. publicly traded), team owners ultimately control central facets of their business and can play a significant role in key strategic decisions. While the ultimate goal of professional team ownership is to be profitable or to maximize winning opportunities depending on the regional or cultural context of professional sport leagues (Watson, 2002; O'Reilly, 2019), a growing chorus of professional sport leaders also have called for a greater focus around social responsibility. In this context, CP in professional sport has become an increasingly significant strategic practice (Philanthropy News Digest, 2013; King, 2019).

There has been a growing body of literature exploring factors related to CSR and CP in professional sport—mainly focusing at the organizational level of analysis and on issues related to the strategic value of these practices (Sheth and Babiak, 2010; Babiak and Trendafilova, 2011; Hovemann et al., 2011; Anagnostopoulos et al., 2017). Notably, some scholarship has shown that the charitable giving behavior of professional sport teams varies widely with some teams recognized for their generosity and involvement while other teams give little or have limited engaged in CP (Inoue et al., 2011; Inoue and Kent, 2013; Sparvero and Kent, 2014; Yang and Babiak, 2021a). There are currently few explanations for these differences. To the best of our knowledge, no scholarship has looked at the influence and involvement of top management or ownership on the role and impact of social responsibility or CP in the unique context of professional sport as one potential explanation for this variance. Given the influence and power of team owners, we argue that examining the relationship between owner characteristics and attributes and the philanthropic giving of their team may offer insights into this variance. The purpose of this study is to explore individual level that might account for the divergence in philanthropic activity in professional sport in the United States. Specifically, we investigate personal/individual owner characteristics and how they might influence organizational outcomes such as charitable giving levels. We draw on conceptual underpinnings from the upper echelons theory, stewardship theory and the organizational governance literature to enhance our understanding of the influences and characteristics of owners on their team's corporate philanthropy efforts and impact.

## Literature Review and Theoretical Framing

### Overview of Corporate Philanthropy

The practice of CP is now widespread globally with charitable giving being practiced by large multinational corporations as well as small and medium-sized enterprises (Gautier and Pache, 2015). While some may view CP as an oxymoron [i.e., “giving money away contradicts the commercial, profit-making purpose of a company” (Gautier and Pache, 2015, p. 346)], Porter and Kramer (2002), argued that engagement in CSR and CP can provide a means to both serve society and contribute to competitive advantage for a business. The scholarly literature on CP has sought to shed light on numerous issues related to CP, from its meaning and essence, to understanding factors affecting resource allocation for philanthropic purposes (Hess et al., 2002; Seifert et al., 2004; Du, 2017), to how CP is structured and organized within businesses (Bruch and Walter, 2005; Brammer and Millington, 2006; Maas and Liket, 2011), to the social and business benefits of CP (Godfrey, 2005; Wang et al., 2008).

Several perspectives can help to frame the behaviors of firms engaging in CP. CP may be viewed as means to serve the common good (Gautier and Pache, 2015) where selflessness plays a role given that these actions are enacted without expectation of any return (non-reciprocity) and the outcomes of CP have been shown to be uncertain and difficult to measure. Given that CP can be considered to fall under the “discretionary” category of CSR (Carroll, 1979) in that it is neither expected nor required, CP may thus be potentially more influenced by the intentions and desires of top executives or owners (Buchholtz et al., 1999). Another perspective views CP as a potential strategic (indirect) investment into the community in which a firm operates (Gautier and Pache, 2015). Investments into the critical needs of a community can bring about strategically important benefits including stronger social capital, enhanced health and safety, more educated citizens/workforce, or improved local services and infrastructure. The argument for a community investment perspective posits that strong social conditions can provide a better work environment for businesses which is central to an organization's competitive advantage (Porter and Kramer, 2002). Thus, decisions around how to deploy CP can provide a firm with strategic benefits which corporate decision makers may wish to leverage and maximize.

CP may be deployed in various ways. Direct donations or corporate giving programs (in the form of money or gifts in kind [product, technical expertise or knowledge, employee volunteerism, etc.)] from a company to a cause (a local or global charity or non-profit organization) is one model of engagement. Another common approach is for CP to be delivered via an associated corporate charitable foundation. According to Giving USA (2021), approximately 4% of total annual giving in the United States ($471 billion)—comes from corporate foundations ($16.9 billion). These are pseudo independent formalized entities that can provide strategic value to a company (through branding, image enhancement, tax relief, etc.) but that operate at somewhat arm's length. In some cases, corporate foundations obtain their grant making funds from direct contributions (endowment) from the profit making entity itself (generally termed as a “Private Foundation” according to the IRS). A different structural categorization of corporate foundations are “Public Charities” which solicit support from the public in the form of grants or donations from individuals or other foundations. Most corporate foundations are private—as many do not solicit or receive public support (Tremblay-Boire, 2020). As both private and public corporate foundations are governed by the US tax code (501(c)3) and are considered “tax exempt,” they must serve the public interest and make their tax filings publicly available (Tremblay-Boire, 2020).

### Professional Sport and Philanthropy

Although the notion of CSR in general has attracted significant interest in both scholarship and practice in the field of professional sport (Walzel et al., 2018), and despite the growing prominence of CP in practice in professional sport (King, 2019), very little research has explored philanthropy, corporate giving, or corporate foundations as a specific research topic in this context. CP is a crucial means for sport teams to engage with local communities, perform civic duties within their communities, and further, foster loyalty and connections with key stakeholders such as fans, youth, businesses, non-profit organizations, and local governments (Kihl et al., 2014). Today, most professional sport teams have a recognized team-affiliated charitable foundation, dedicated to raising money to give away to local (non-profit) partners or to support pressing social issues in communities where they operate (Babiak and Wolfe, 2009; Walters, 2009; Sparvero and Kent, 2014; Yang and Babiak, 2021a).

Of the published research in this area, some studies have investigated the relationship between CP levels and team/organizational factors. For example, Inoue et al. (2011) investigated the relationship between team charitable giving and team financial performance and found there was no relationship between being socially responsible and team financial performance. The authors argued that the findings may indicate that team foundation activities and team operations might not be fully strategically integrated. In another study examining professional team foundations, Sparvero and Kent (2014) analyzed team foundation efficiency using 8 years of charitable financial reporting data. They found significant variance among teams and leagues related to average annual giving depending on how an organization is classified (i.e., a public charity or private foundation). They also demonstrated the growth and emphasis of team foundation mission-related spending over the 8-year period of their investigation suggesting that the role of CP is becoming increasingly relevant in professional sport. In addition, some recent studies have begun to explore how the level of giving by team foundations is influenced by the institutional environments of professional sport teams, such as institutional peers (e.g., the influence of sport teams in the same sport league or local sport teams from different sport leagues) (Yang and Babiak, 2021a). This work has found that teams were more likely to be attentive to their league peers than local peers. In addition, Yang and Babiak (2021b) explored how institutional pressures from the community in which the teams are located promote or constrain team's philanthropic giving, and uncovered that higher state income tax rates and a greater presence of nonprofits in the community increase teams' philanthropic giving.

While this research has contributed to a deeper understanding of the strategic role that CP can play for professional sport teams, the studies in this area have focused primarily on the institutional or organizational levels of analysis. We still know little about how individuals can influence or impact the extent of philanthropy of professional sport teams, in particular, how do owners affect and shape efforts around CP in their own teams?

### Team Owners, Philanthropic Determinants, and Corporate Philanthropy

Significant attention has been paid in the literature to the drivers of philanthropy in business, seeking to understand why companies might make voluntary contributions to serve purposes beyond profit maximization (Brammer and Millington, 2006; Marquis and Lee, 2013). Much of the literature has explored the growing pressures for social engagement from external (political or social) stakeholders (Brammer and Millington, 2006). However, since “...organizations are social entities where decisions are made by actors with various interests” (Gautier and Pache, 2015, p. 349), looking inside the organization to understand philanthropic drivers is critical. Importantly, understanding owner and top manager influence can “...help to shine a light on the internal contingencies by which philanthropy may arise as a business strategy or as an agency loss” (Marquis and Lee, 2013, p. 484). Many of the existing studies seeking to understand the relationship between top management and philanthropy have been carried out exploring CEO and top management team characteristics in publicly traded companies and recently in emerging markets such as China (Manner, 2010; Marquis and Lee, 2013; Wei et al., 2018). However, limited research has focused on the ownership of a firm as a potential predictor of its charitable behavior, particularly in privately owned companies (Campopiano et al., 2014). Owners of privately owned firms may have more discretion and power to influence decisions and choices around CP.

The nature of professional sport team ownership is rather unique and has seen numerous changes over the past two decades (O'Reilly, 2019). As professional sport team values have appreciated and revenues have increased, the landscape of team ownership has shifted to include primarily affluent individuals and families, partnership groups, and/or corporations (Badenhausen, 2020). While some acknowledge that the primary purpose of owning a team is profit seeking, there are some team owners who may focus on non-financial goals and use the team to help leverage alternate business, social, or personal objectives (Foster et al., 2016). For instance, some owners spend significant amounts on star player salaries which may reduce profit for the sake of winning on the field (O'Reilly, 2019). Owning a team may be a status symbol for some owners to enhance their image, reputation and prestige, and for others, team ownership may also reflect a commitment to protecting a community asset (Foster et al., 2006). Through CP, owners may satisfy their altruistic values or other strategic aims by influencing the levels of philanthropic giving to the community where the team operates (Inoue et al., 2011).

In professional sport, team owners have decision-making discretion in establishing the organizational structure and chain of command as well as strategic development, implementation and assessment—including around choices related to CP (Robinson, 2005; Carter, 2015; Juravich et al., 2017; O'Reilly, 2019). Professional sport team owners have been described as deeply and personally committed to ensuring that they and their team contribute beyond simply sport performance. “They are philanthropists who see an opportunity to extend their personal commitment to make a difference.” (Hohler, 2005, D1). Some team owners have made public statements about their CP aims and intentions, suggesting that they are key influencers in strategic directions such as cause support, structure and design, and goals and objectives of their team's charitable efforts (Robinson, 2005). For example, the owner of the Golden State Warriors of the NBA, Joe Lacob stated:

“*When we bought the team in 2010 we wanted to have an impact on the community. As an organization you can do that in a variety of ways. All my philanthropic ventures prior to the Warriors generally involved education. So we decided to choose that as (the focus) of the Warriors Community Foundation. I want it to be the largest foundation in the NBA…. and in the Bay Area. We can do it… it's going to grow. We've got real plans for it” (Murray*, *2018**)*.

Another owner group, the DeVos family, has also prioritized a focus on philanthropy as noted in the following statement:

“*When the DeVos Family purchased the [Orlando] Magic, [owner Rick DeVos'] vision was that the team and organization would serve as a platform to improve the Central Florida community. That legacy will certainly live on in the Orlando Magic's community efforts and philanthropic contributions" (NBA Media Report*, *2018**)]*.

Thus, it is clear that professional sport teams, given their unique ownership structures as private businesses and the tensions between winning / profits, and other motives for team ownership (O'Reilly, 2019) provides a rich context in which to explore the drivers of CP.

### Individual Level Theoretical Approaches to Corporate Philanthropy

While there are numerous forces that can drive CP, in this paper we explore the role of individuals as decision-makers and proponents in this domain. As Buchholtz et al. (1999) noted “...[CP] is consistent with virtue ethics, a system of thought that focuses on the quality of an individual's character. Although corporate philanthropy is an organizational action, it is prompted by the decisions of individual managers.” (p. 169). To help unpack our understanding of individual aspects shaping CP in a firm, we draw on an upper echelons perspective to provide richer insights into facets of team ownership and philanthropy. The upper echelon's view posits that a relationship exists between characteristics of leadership/top management and organizational outcomes (Hambrick and Mason, 1984). One of the central premises of upper echelons theory is that the experiences, backgrounds, values and other (individual) factors influence interpretations of the situations that leaders or executives face, and in turn, ultimately affect the choices/decisions they make (Hambrick, 2007; Juravich et al., 2017; Wei et al., 2018). Upper echelons research has uncovered the effects of executive characteristics on the strategic decisions of firms (Petrenko et al., 2016). According to this approach, although many individual attributes such as a person's values, beliefs, or cognitions may be difficult to measure or assess, other observable executive characteristics—such as demographic features—can be used as a proxy which can then help predict organizational strategy and outcomes (Hambrick, 2007; Wei et al., 2018). We also consider elements of organizational identity theory in this study which is another theoretical framework often used in research exploring family businesses that may inform a deeper understanding of team owner interest around CP (Dutton and Dukerich, 1991; Bingham et al., 2011). This theory suggests that CP actions may serve personal instrumental utilities in that individuals who are closely linked to a business are more likely to emphasize corporate reputation, as it is perceived to be associated with their own individual image and reputation (Zellweger et al., 2013). Next, we hypothesize how owner characteristics and attributes affect the philanthropic giving of their team.

### Ownership Characteristics and Corporate Philanthropy

Previous studies into philanthropic behavior have identified a number of general demographic features associated with an individual's propensity to give. For example, older people seem more inclined to donate than younger people, women tend to give more than men, and individuals with higher education levels are more likely to donate to charity (Sargeant, 1999; Nichols, 2000; Yao, 2015; Bjälkebring et al., 2016). As some of the above studies have suggested, it may be possible to find a link between individual philanthropic activity and certain personal idiosyncratic features. An owner's stake in the organization, philanthropic experience, age, tenure of ownership, education level, and connection to community may be indicators of social concern, and we hypothesize that it would appear likely that such factors might also be indicative of levels of professional sport team philanthropic contributions.

#### Ownership Structure

The level of power and control an owner has may not only shape their relationship to their business, but also impact their potential to influence organizational decisions (Gao et al., 2017). Since power in decision making may be determined by the ownership level, we expect that the ownership structure of the team may impact levels of interest and engagement in CP. Professional sport teams have a range of ownerships forms, with some teams having an individual with majority control of the team and other teams operating in a partnership or syndicate structure (O'Reilly, 2019). For some professional teams with an individual majority owner, this person may have the primary responsibility to make major decisions for their team, and thus team CP decisions may reflect their interpretation of perceived pressures, and/or their own values, attitudes and priorities toward CP (Gao et al., 2017). In teams with widely dispersed ownership shares (like partnerships, syndicates, or investment group structures), multiple owners may be less likely to have discretionary influence over CP. As O'Reilly (2019) noted, “A syndicate is inherently more complicated to govern than a single owner model as individual members will have different priorities, voting rights, decisions rights and roles to occupy” (p. 134). In these cases, decisions and discretion around corporate giving and activities engaged in CP may be left to other (team) organization executives who may be more cautious or conservative about deploying company resources (Adams and Hardwick, 1998).

Haley (1991) found that greater discretion to make charitable and other donations could help business owners increase their own prestige in the local community and thereby enhance the value of their reputational capital. Gallo (2004) and Miller and Le Breton-Miller (2005) also found that individual or family owned companies have a greater willingness to develop connections with stakeholders and act as good stewards of the communities in which the business operates through philanthropy. In individual ownerships structures, individual and business rationales are closely intertwined. Often, philanthropy is seen as a way of achieving business goals and supporting the company (team) and its stakeholders (Andreoni, 2006). As such, individual owners may be more likely to be concerned about firm philanthropy, to nurture personal relationships with external stakeholders and, generally, to behave as good community stewards. Thus, owners who are proud of their business and are willing to enhance its reputation by contributing to the community consider firm philanthropy to a greater extent (Litz and Stewart, 2000; Miller and Le Breton-Miller, 2005). Therefore, we propose:

**Hypothesis 1**: *Professional sport teams with individual owners are likely to have larger charitable giving than teams with a partnership / investment group ownership structure*.

#### Owner Previous Philanthropic Involvement

The ethical and moral values of a firm's owners and leaders may drive decisions to engage in giving behavior (Galaskiewicz, 1985; Valor, 2006). Senior executives and owners may influence choices around their firm's philanthropy following their own attitudes and beliefs toward charitable giving, their ethical compass, and their personal values and integrity (Choi and Wang, 2007). This individual pro-social perspective was supported by a study carried out by Dennis et al. (2009) who found that a critical driver of a company's philanthropy was the extent to which the top manager identified themselves as a philanthropist. Thus, previous altruistic acts of owners may influence CP choices made through their own firm.

Cha et al. (2019) supported this view and argued that CEOs' previous civic engagement positively impacts a firm's CSR efforts. The upper echelon theory posits that business leaders tend to focus on external social and environmental issues that align with their personal and professional experiences and cognitive frames (Cha et al., 2019). Their study, based on tenets from upper echelon theory, examined how ideology, personal knowledge, life experiences and personal civic and social engagement may play an important role in how a CEO shapes the level of a firm's CSR involvement. Personal convictions and passions around making a positive impact on society can influence business leader's commitment to support meaningful social causes through their firm (Marquis and Lee, 2013). Cha et al. (2019) found that the personal level of CEO social engagement was “a significant positive predictor of corporate philanthropy” (p. 1,062). From this, we extrapolate that high levels of previous social engagement may influence professional sport team owners to project their own experiences, values, and beliefs around their team's CP efforts. Therefore, we expect that team owners who have demonstrated previous levels of personal social engagement—through their own philanthropic actions—will have higher levels of CP giving. Based on these arguments, we propose:

**Hypothesis 2**: *Professional sport teams with owners who have previous philanthropic experience will have larger charitable giving*.

#### Owner Tenure

The length of time an individual has owned a team may influence their choices around their firm's philanthropy. Upper echelon research has demonstrated that there are changes in CEO behaviors and priorities over the course of their tenure in an organization (Wei et al., 2018). Newer team owners may want to legitimize themselves in a community where they have recently purchased a team by showing strong engagement in CP (Marquis and Lee, 2013). In doing so, they may seek integration into important stakeholder networks and establish and signal their commitment to the community (Simsek, 2007). Newer team owners may also seek avenues such as CP to build coalitions and community relationships and attract key resources to help plan, develop and support (future) team initiatives such as stadia financing and development, or other strategic team plans (O'Reilly, 2019). As such, newer owners may be more sensitive and responsive to external perceptions and pressures from community constituents such as local governments, media, sponsors and local businesses, and other potential partners, and may be more generous through their team's charitable foundation. Longer term owners may tend to be less innovative and look to peers for cues around CP as they are less likely to effectively match the organization's strategy with the external environmental conditions—that is, they become “stale in the saddle” (Miller, 1991; Marquis and Lee, 2013). Thus, we propose:

**Hypothesis 3***: Professional sport team owners' tenure will be negatively related to charitable giving of the team*.

#### Owner Education

The level of education of senior managers has been found to be an important determinant of individual values, perceptions and cognitions, and influence on firm performance (Hambrick and Mason, 1984; Gottesman and Morey, 2006; Manner, 2010). Wei et al. (2018) suggested that the level of education may impact how an individual perceives and responds to opportunities such as CP. O'Neill et al. (1989) also found that higher education levels are associated with stronger corporate social awareness. Other research suggests that education level is related to moral development and moral reasoning abilities (Jones et al., 1990) as well as open-mindedness and information processing related to decision-making (Hambrick and Mason, 1984). Huang (2013) and Wei et al. (2018) also found that the type of education (graduate level professional programs such as MBA or law degrees) impacts leader decisions around CP given their focus on short-term business objectives such as operational efficiency and immediate profits sometimes at the cost of moral and ethical longer term impacts and outcomes. Thus, we propose:

**Hypothesis 4**: *Professional sport teams with owners who have higher than undergraduate level education will increase their charitable giving*.

#### Owner Age

Owner age may be a predictor of CP. A recent study by Fidelity Charitable (2019), for example, showed a higher tendency for millennial entrepreneurs to engage in charity and philanthropy (via financial donations and volunteerism) than entrepreneurs from previous generations (i.e., Boomer and Gen X). However, previous research on individual giving shows that older people are more likely to give to charity, with individuals over 60 more than three times more likely to give than younger people are. Previous research has shown a positive relationship between age and propensity of charitable giving (Midlarsky and Hannah, 1989; McAdams et al., 1993). For example, Bjälkebring et al. (2016) found that older individuals have a greater level of positive emotional reaction from monetary donations compared to younger adults. The authors argued that an age-related positivity bias influences older people to draw more positive affect from charitable donations than their younger counterparts.

In the context of the influence of CEO characteristics on socially responsible practices, McCuddy and Cavin (2009) noted that older CEOs have a stronger motivation to “give back” to their communities because they are more likely to have servant leadership styles (empathy and stewardship) due to the accumulation of social expertise and greater cultural intelligence (Hess and Auman, 2001; Shannon and Begley, 2008). Furthermore, Ng and Sears (2012) found that age is a significant moderator that influences CEOs to implement organizational diversity practices (e.g., diversity programs related to Equal Employment Opportunity and Affirmative Action). Given that older CEOs may be more inclined to be concerned about CP/CSR, we postulate that the age of professional sport team owners will be positively related to their team's propensity to donate to charitable causes.

**Hypothesis 5**: *Professional sport team owners' age will be positively related to the charitable giving of the team*.

#### Owner Connection to Community

Foster et al. (2016) argued that professional sport team owners display a wide array of motivations for owning the team. The authors noted that owners' passion for sport and love for the team or hometown/community in which it operates is one such motivation. Team owners may have a connection to the community where their teams are located as they either come from the community themselves or operate their business in the same region. Foster et al. (2016) also suggested that team owners try to enhance their community and national profile through owning a sport team. Specifically, Atkinson and Galaskiewicz (1988) noted that CP could be a strategic decision of executives to “gain approval and respect from local business elites” (p. 82).

Meanwhile, professional sport teams and family firms may have a lot in common in terms of their governance and ownership structure. Both types of organizations are privately held—where owners (or their family members) maintain significant control over the organization. Within family business literature, scholars have argued that family firm owners perceive themselves as a steward of the community in which the firm operates as it is crucial to develop a connection with stakeholders in the surrounding community (usually the primary business market) (Miller and Le Breton-Miller, 2005; Bird and Wennberg, 2014; Campopiano et al., 2014). In addition, strategic philanthropic initiatives can not only serve the community's needs but also preserve a competitive environment to do their business (Gautier and Pache, 2015).

The primary residence of team owners may represent the extent to which owners have a connection to the community. That is, it may suggest that owners might be more sensitive to community needs or that they may have a greater “local” understanding of corporate philanthropy. Assuming that the primary residence of the team owner represents the owner connection to the community, we propose that

**Hypothesis 6**: *Professional sport teams with owners who live in the communities where their team is located will have larger charitable giving*.

## Methods

### Data and Sample

To empirically test the hypothesized owner characteristics on CP outcomes, we gathered data on professional sport team foundations and each team owner. Our primary data source was team foundation charitable giving data from Internal Revenue Service (IRS) Form 990s. We examined four sport leagues in the U.S (i.e., National Basketball Association [NBA]; National Football League [NFL]; National Hockey League [NHL]; Major League Baseball [MLB]). Nonprofit (501(c)(3)) organizations file 990 annual tax reports to the IRS. These reports contain financial information, such as revenues (e.g., contributions and grants received), expenses (e.g., grants and program-related spending) and assets. Form 990s were collected from Candid, a database that compiles financial and organizational information for all American non-profit and charity organizations. Team owner data were manually collected from multiple sources, such as the official websites of professional sport teams, team owners' corporation websites, other databases (e.g., Statista, Sports Reference.com) and media reports (e.g., Forbes Magazine, Business Insider). Other team-related data were collected from ESPN (e.g., home game attendance percentage), Forbes's annual financial reports of U.S. professional sport teams, Rodney Fort's Sport Business Database (e.g., team revenue and expenses), and the official website of each professional sport league (e.g., teams' regular season winning percentage). Community demographic data (e.g., identification of team-located metropolitan statistical area [MSA] and local population / income) were gathered from the U.S. Census Bureau.

Our sample included 760 team-year observations from 94 U.S. professional sport teams (i.e., 26 MLB teams, 22 NBA teams, 27 NFL teams, and 19 NHL teams) during the period between 2009 and 2017. This sample includes professional sport teams with an affiliated charitable foundation (either public charity or private foundation). Although most sport teams have formed a recognized foundation, some teams have utilized other forms of organization as their philanthropic arms, such as a private (team owner) family foundation, entertainment company foundation, or local community foundation. Such cases were excluded from the sample. Moreover, sport teams owned by corporations were excluded from our sample, as they are not suitable for testing our hypothesized team owner's effect on CP.

### Measures

#### Dependent Variable

Our dependent variable, *team philanthropic giving*, was defined as the sum of the total amount of grants and program service expenses by a team foundation for each year. This variable captured both the direct cash/grants made to charities and individuals as well as the indirect philanthropic contributions team foundations made through their direct service programs (e.g., community development programs, after school programs, special events). The dependent variable was log-transformed to correct for skewed values.

#### Independent Variables

*Individual ownership* represents a professional sport team owned by a single owner who possesses the majority of equity of the team (O'Reilly, 2019). Team owner *personal foundation* is a dummy variable indicating whether the team owner has established his/her own charitable foundation, representing the owner's previous philanthropic involvement. Team owner *tenure* represents the number of years the owner has owned the franchise. Team owner *higher education* indicates the educational attainment of the team owner (i.e., completion of a graduate degree or above [e.g., master's degree, MBA, law degree or other doctoral degree]). Team owner *age* represents the chronological age of the owner in the given year. Finally, team owner *community residence* is a dummy variable representing whether the owner resides in the community (Metropolitan Statistical Area [MSA]) in which the team is located.

#### Control Variables

First, we included a dummy variable for *multiple team ownership*, indicating whether the team owner owned more than one professional sport team across sport leagues. We included several control variables at the team foundation and team level to control for factors that might affect CP. On the team foundation level, specifically, *private foundation* represents whether the team foundation was classified as either a private foundation or a public charity according to IRS denotation. Some research has found that team foundations designated as public charities tend to have higher program service expense ratios than those of private foundations (Sparvero and Kent, 2014). *Foundation revenue* and *foundation asset* were measured by the total annual revenues and assets of the foundation in the year of interest, respectively.

We also controlled for team level factors such as *team revenue* (measured through annual team income [e.g., revenues from ticket sales, local and national media broadcasting rights, sponsorship, concessions, merchandise]); *team operating efficiency* (the proportion of operating income over total annual revenues (Inoue et al., 2011), representing the team's financial performance); *team age* (logged); and team *winning percentage* (the percentage of regular-season games won by each team [Foster and Washington, 2009]); and, team *attendance percentage* (the annual home game attendance as percentage of stadium capacity reflecting actual purchasing behaviors of sport fans (McDonald and Rascher, 2000; Inoue et al., 2011).

Furthermore, we controlled for demographic characteristics of the community in which team foundations operate based on MSA [i.e., the region that consists of the city and surrounding community which typically have a high degree of social and economic integration (U. S. Department of Commerce, 2010)]. We collected data on *local population* and *local income* (i.e., per capita income of the community). These variables capture the overall economic situation and the size of the community in which teams operate (Marquis et al., 2013).

We utilized consumer price index (CPI; U. S. Bureau of Labor Statistics, 2022) to adjust inflation and capture the real dollar value (2009 as a base year) for t*eam philanthropic giving, foundation revenue and asset, team revenue, team operating efficiency, and local income*.

### Empirical Approach

To test the hypotheses with the panel data, we used a fixed effects model to account for multiple observations per team. We performed a series of tests to determine whether to consider team effects using fixed or random effects in our model. First, we conducted an F-test of the fixed effects model. We found that the null hypothesis that all fixed effects were jointly 0 was rejected, which provided evidence that a fixed effects model was preferable to a pooled ordinary least squares (OLS) model. Second, the Breusch-Pagan Largrange Multiplier (LM) test for testing random effects was conducted. We found that the random effects model was more efficient than the pooled OLS model. Finally, we performed the Hausman test to determine whether the fixed effects model was more appropriate for the analysis. The test rejected the null hypothesis that the unique errors (i.e., fixed effects) were not correlated with the regressors. Thus, we concluded that a fixed effects model was preferable in the analysis (Hausman, 1978).

Additionally, we inspected multicollinearity by calculating the variance inflation factor (VIF) based on the fully specified model. The VIFs for each independent variable were below 10 (ranged from 1.10 to 2.50), and thus it suggested that the models had no multicollinearity issues (Neter et al., 1985). We used standard errors robust to heteroskedasticity in all models.

## Results

Table 1 displays descriptive statistics and correlations coefficients of all variables. As seen in Table 1, the average annual team philanthropic giving was about $1.1 million. Approximately 70 percent of teams were owned by a single owner. Team owner tenure ranged from 0 to 55 years with an average of 13.6 years. On average, team owners were 64 years old and about 44 percent of them have completed a graduate degree or above. The percentage of team owners who have personal foundation and live in the same community where the team located were ~44 and 66 percent, respectively.

**Table 1:** Descriptive statistics and correlations.

**Variables**	**Mean**	**SD**	**1**	**2**	**3**	**4**	**5**	**6**	**7**	**8**
1. Philanthropic giving	1.11	1.15								
2. Individual ownership	0.689	0.463	−0.071							
3. Personal foundation	0.437	0.496	−0.062	0.144						
4. Tenure	13.65	11.43	0.001	0.120	0.118					
5. Higher education	0.439	0.497	−0.003	−0.133	−0.064	−0.099				
6. Age	64.37	12.34	0.047	0.098	0.137	0.526	−0.056			
7. Community residence	0.664	0.472	−0.012	−0.025	0.103	0.063	0.090	−0.020		
8. Multiple team ownership	0.199	0.399	−0.115	0.185	0.027	0.105	0.071	0.129	0.172	
9. Private foundation	0.211	0.408	−0.056	0.158	0.014	−0.010	0.063	0.040	−0.071	0.091
10. Foundation revenue	1.36	1.36	0.712	−0.153	−0.048	−0.041	−0.059	0.009	0.010	−0.016
11. Foundation asset	1.76	2.20	0.417	−0.104	−0.014	0.002	0.030	0.090	0.119	0.108
12. Team revenue	213.9	100.3	0.348	0.148	−0.073	0.164	−0.034	0.106	0.016	0.111
13. Team operating efficiency	0.104	0.128	0.208	0.067	−0.088	0.208	−0.084	0.108	0.156	0.150
14. Team age	49.44	32.80	0.353	−0.162	−0.202	0.137	0.175	0.030	−0.056	−0.015
15. Attendance percentage	87.33	16.07	0.028	0.093	0.075	0.051	0.149	−0.019	0.082	−0.016
16. Winning percentage	0.506	0.141	0.117	−0.074	0.016	0.034	0.078	0.022	0.068	−0.061
17. Local income	29.83	4.70	0.166	−0.101	−0.013	0.016	−0.018	−0.073	0.171	0.134
18. Local population	5.89	5.10	0.106	0.079	0.123	−0.112	0.075	−0.023	0.069	−0.116
**Variables**		**9**	**10**	**11**	**12**	**13**	**14**	**15**	**16**	**17**
10. Foundation revenue		−0.055								
11. Foundation asset		−0.005	0.633							
12. Team revenue		0.095	0.356	0.281						
13. Team operating efficiency		0.103	0.187	0.199	0.579					
14. Team age		−0.056	0.311	0.317	0.347	0.194				
15. Attendance percentage		−0.109	0.125	−0.018	0.172	0.247	−0.082			
16. Winning percentage		−0.095	0.125	0.049	0.019	0.039	0.001	0.183		
17. Local income		0.134	0.221	0.151	0.312	0.207	0.173	0.034	0.123	
18. Local population		0.078	0.060	0.109	0.190	−0.021	0.180	0.041	0.019	0.178

*This table reports means and standard deviations using the original untransformed variables (Philanthropic giving, foundation revenue, foundation asset, and team revenue in millions of dollars; team age in years; local income in thousands of dollars; local populations in millions)*.

Table 2 presents the results of the fixed effects model estimating the effect of owner characteristics on team philanthropic giving. Model 1 estimates the coefficients of our control variables. Model 2 presents all main effects of team owner characteristics. The results show support for Hypotheses 2, 3 and 5. Hypothesis 1 suggested that professional sport teams with individual owners would be more likely to have larger charitable giving than teams with a partnership/group ownership structure. However, our analysis did not find support for H1. Hypothesis 2 predicted a positive relationship between team owners' previous philanthropic experience and philanthropic giving, and Model 2 strongly supports this prediction. It was estimated that, on average, teams with owners who have a separate personal foundation had an approximately 34 percent increase in team philanthropic giving (it was assumed that one-unit change in the independent variable was associated with 100·β1 percent change in the dependent variable in the interpretation of coefficients in log-linear regression). Hypothesis 3 proposed a negative association between owner tenure and team philanthropic giving. Model 2 supported this hypothesis. Specifically, on average, a 1-year increase in owner tenure is associated with an approximately 2.6 percent decrease in team philanthropic giving. Although the higher educational level of team owners was significantly associated with greater team philanthropic giving, our additional model for robustness check failed to support Hypothesis 4. We found that a positive relationship exists between team owner age and the level of philanthropic giving. In particular, the results suggested that, on average, a 1-year increase in owner age is associated with an approximately 1.9 percent increase in team philanthropic giving. Finally, Hypothesis 6 suggested that team philanthropic giving would be greater when team owners reside in the community where the team is located. Our model did not support H6.

**Table 2:** Effect of owner characteristics on team philanthropic giving.

**Variables**	**Model 1**	**Model 2**
Individual ownership (H1)		0.058
		(0.227)
Personal foundation (H2)		0.337*
		(0.173)
Tenure (H3)		−0.026**
		(0.008)
Higher education (H4)		0.372*
		(0.193)
Age (H5)		0.019*
		(0.010)
Community residence (H6)		−0.105
		(0.259)
Multiple team ownership	−0.293	−0.381^+^
	(0.316)	(0.228)
Private foundation	−0.179	−0.233^+^
	(0.120)	(0.120)
Foundation revenue	0.283***	0.282***
	(0.049)	(0.053)
Foundation asset	−0.050^+^	−0.044*
	(0.025)	(0.022)
Team revenue	−0.002	−0.002
	(0.001)	(0.001)
Team operating efficiency	0.168	0.138
	(0.349)	(0.333)
Team age	0.063**	0.061**
	(0.022)	(0.021)
Attendance percentage	0.394	0.293
	(0.433)	(0.385)
Winning percentage	0.207	0.137
	(0.186)	(0.177)
Local income	0.044	0.044
	(0.042)	(0.042)
Local population	−0.269	−0.079
	(0.280)	(0.242)
Constant	10.272***	8.339***
	(1.621)	(1.567)
Observations	760	760
Number of team	94	94

*Robust standard errors in parentheses*.

*All models include team and year effects*.

+ *p <0.01*,

* *p <0.05*,

** *p <0.01*,

*** *p <0.001 (One-tailed for hypothesized, two-tailed for controls)*.

We conducted additional analyses by including squared terms of age and tenure to examine the quadratic relationship between age/tenure and philanthropic giving. The new model including these squared terms yielded results similar to our original model. Additionally, we found that team philanthropic giving increases with owner age at a decreasing rate (i.e., the positive linear age term and negative squared age term), which might show a more nuanced relationship between age and philanthropic giving. The squared term of owner tenure was not significant. In addition, we analyzed alternative models by specifying the dependent variable differently. Specifically, the model used the alternative dependent variable, the proportion of philanthropic giving over team revenue (while removing team revenue from the control variable). We believe that this additional analysis served as robustness check by providing further substantiation of our results. Although the results using this ratio variable were similar to those presented above, it did not support the positive relationship between owner educational level and team philanthropic giving.

## Discussion and Conclusions

Our study focused on which team ownership characteristics influenced corporate giving levels of professional sport teams. To this end, we analyzed a longitudinal data set of U.S. professional sport team foundations and team owners. A number of attributes of team ownership—including previous philanthropic involvement, owner tenure, and owner age—were shown to influence a team's philanthropic giving. However, the effects of owner structure, education level, and community residence on philanthropic giving were not found.

Our findings did not find a significant effect of ownership structure on a team's charitable giving. We expected that power and control would enable individual owners to enact decisions regarding (increased) philanthropy levels. It appears that other factors might determine professional sport team owners' decisions and involvement in CP and the business agenda rather than individual image, prestige and charitable reputation building. Indeed, corporate donations through team foundations may benefit all stakeholders, partners, investors and owners (individual, institutional etc.) and owners may view CP as more cost-effective as a company expense rather than separate individual out of pocket contributions (Adams and Hardwick, 1998). Also, it would be interesting for future research to explore how family team ownership (e.g., Dallas Cowboys—Jones family, New England Patriots—Kraft family) might influence on CP. Regarding the effect of owner education levels on a team's charitable giving, we found a positive relationship from our main model; however, our robustness check did not support this result. We believe this inconclusive result suggests that investigating educational level would be a broad-brush attempt to explore such relationship, and it is plausible that their academic field could be a more relevant factor to CP rather than an owner's education level. For example, Manner (2010) found that CEOs having a bachelor's degree in humanities is positively related to corporate social performance (CSP), while a bachelor's degree in economics is negatively related to CSP in the short term. Finally, owner connection to community was also not found to have a significant effect on team CP. These findings implied that the residence of team owner is not necessarily associated with the level of philanthropic giving by the team. Team owners are typically wealthy individuals who have accumulated wealth from their business success. It is plausible that they operate business(es) across the country, and thus have connections to multiple communities. Although some team owners, especially when they are originally from the community in which their teams are located, may have owned a team purposely to keep the team in his or her community and prevent relocation (O'Reilly, 2019), other team owners might have varied ownership intentions, such as enhancing personal profile to boost their business operations. If that is the case, team owners may be less likely to see themselves as stewards of the community or want to build connection to community compared to other types of family business owners (Campopiano et al., 2014).

Our study did uncover three significant individual level owner attributes influencing CP in professional sport teams. Our findings suggested that professional sport teams with owners who have a personal foundation have a higher propensity to donate to charitable causes. Although CP might be a part of firm strategies related to enhancing firm reputation, generating economic returns, and improving stakeholder management (Wang and Qian, 2011), it was noted that philanthropic decisions are often based on “beliefs and values” of top executives (Porter and Kramer, 2002, p. 6). There has been past research that shows the positive relationship between CEO's altruism, civic engagement (e.g., fundraising, volunteering, or work with non-profits) and a firm's CSR efforts (Waldman et al., 2006; Cha and Rew, 2021). Similarly, Choi and Wang (2007) found that firm leaders with benevolence and intrinsic concerns for others were more likely to be involved in corporate philanthropy. It may be the case that previous experience in philanthropy or involvement with individual foundations may affect team owners in cultivating organizational/team values and priorities around CP.

We also found that team owner tenure is negatively associated with the level of team philanthropic giving. One explanation for these findings is that team owners in their early years may aim to increase their reputation and build community relationships through CP. That is, a reputation for CP can support protecting firm relationships with their stakeholders and decrease a firm's risk of losing critical resources (Godfrey, 2005). Moreover, Wei et al. (2018) suggested that it is plausible that executives with shorter tenures may be prone to donate more and broadcast their donations so that they can nurture stakeholders' relationships to countervail uncertainties in the given institutional environment. Given that sport teams are closely tied to a specific city or community (and fans), philanthropic involvement in their communities may help in improving public relations benefits, and enhancing loyalty and connections with local fans (Babiak and Wolfe, 2009; Yang and Babiak, 2021a). Specifically, newer team owners may be more likely to exhibit a passion for CP so they can generate reputation and goodwill among stakeholders of teams (Brammer and Millington, 2005; Ling et al., 2007).

Finally, there was also a significant relationship between owner age and the level of a team's philanthropic giving. This positive relationship between age and philanthropic giving is supported by previous research (c.f., McAdams et al., 1993; McCuddy and Cavin, 2009). In a meta-analysis of research on prosocial/altruistic behavior, Fabes and Eisenberg (1996) found that prosocial behaviors such as charitable giving tended to increase with age. Older CEOs/owners may be more interested in CP given pressing concerns to imbue values and norms into the organization before they hand over the reins to new owners (Asfshar, 2012). Furthermore, our additional analysis demonstrated the quadratic relationship between owner age and philanthropic giving (i.e., an inverted U-shape). In other word, philanthropic giving increases in age at a decreasing rate, which suggests that the positive effect of age on philanthropic giving is greater when team owners are relatively young.

This study sheds light on how ownership characteristics can affect levels of professional sport team CP and fills a gap in the literature by connecting individuals to CP related outcomes (Orlitzky et al., 2011). The findings from this research shed light on the important role of owners in shaping the levels of a team's philanthropic contributions, community support, and engagement. Furthermore, given that much of the research on corporate foundations has been qualitative and focused on individual foundations (Tremblay-Boire, 2020), this research contributes to the literature by providing insights into a subset of unique corporate foundations in the professional sport context in the United States.

As Wood (1991) noted “…the business and society field has not built a concept of discretion, or discretionary social responsibility… A company's social responsibilities are not met by some abstract organizational actor; they are met by individual human actors who constantly make decisions and choices, some big and some small, some minor and others of great consequence” (pp. 698–699). Given that society demands greater accountability of business in creating meaningful social impact, the role of business leaders would be crucial in shaping the scope and impact of their organization's social outcome. In professional sport, team owners are symbolic figures in their organizations and can exercise their philanthropic leadership and represent individual and team values to external stakeholders. By highlighting the critical role, power, and influence of team owner, this study further suggests that certain individual characteristics of organizational leaders can be useful indicators to understand the socially responsible behavior of professional sport teams.

### Practical Implications

The findings from this research offer a number of practical insights for advancing the practice of professional sport team philanthropy. Being attuned to the interests, values, and pro-social aims of top organizational leaders can cue team foundation executives to not only the team's charitable expectations in terms of the amount of giving, but can also inform the focus and direction of philanthropic efforts (i.e., the types of causes to support, specific groups of interest). Team owners are also influential actors in communities, and through team CP, they can amplify the focus on pressing social causes in cities where their teams exist, potentially leading to more positive social outcomes. The insights from this study may also be helpful for local non-profits/grant seekers who may adopt appropriate strategies to engage with team foundations by understanding the team owner's motives, values, and interests around CP.

### Limitations and Future Research

This research has established that individual characteristics of professional sport team owners may be one important variable in understanding corporate charitable contributions. However, we believe that there are still many unexplored questions that could help to inform insights into how individuals in businesses might impact CP. Our study examined observable attributes of firm ownership. It may be the case that other individual level factors can influence how and why a business owner might consider charitable action through their firm. For instance, personality characteristics such as hubris, humility, narcissism, or overconfidence (Hayward and Hambrick, 1997; Chatterjee and Hambrick, 2007) have been explored in varied contexts related to other measures of firm performance (e.g., innovation, strategic change, diversification), but not related to CP. In addition, behavioral characteristics such as leadership style or degree of involvement in decision-making may be relevant influences on levels of CP. Other intervening variables which affect the adoption of CP in professional sport could also be identified, such as income/net worth, marital status, gender, sex, religion, number of children, or political party affiliation. Moreover, it would be desirable if future research could examine the functional backgrounds of team owners (e.g., industry sectors of team owners' businesses). These individual level influences on corporate giving would be important to uncover in future research to gain a more nuanced perspective of the variance in CP across sport businesses.

Another significant gap in the literature exists in understanding the decision-making process to determine priorities, strategies, targets, and business activities around CSR and CP in sport. The literature remains unclear on which and how actors influence philanthropy decisions in corporate foundations (Muller et al., 2014). There is still considerable debate in the CP literature around the influence of senior executives (such as firm CEOs or presidents) or senior corporate foundation leaders (i.e., executive director, foundation president) on the direction and level of corporate giving (Tremblay-Boire, 2020). In addition, it would be desirable to examine how professional networks act as conduits through which norms/standards around CP flow and affect the engagement level of a team's socially responsible activities.

Our research did not explore the dynamic between owners and top team management (e.g., CEO/president), where agency theory concerns might be more prevalent and may not only impact the level of giving but the focus and form of charitable behaviors as well. These questions are still unanswered in the sport context and may be interesting to explore given professional sport's unique structure and governance. The influence of stakeholders (such as employees/athletes, community actors, or corporate sponsors) on sport team charitable giving efforts is also an area of opportunity for CSR and CP researchers. Specifically, given the unique context of the current situation (i.e., economic uncertainty, a global pandemic, and social unrest), professional team owners may look to philanthropy as a potential avenue of strengthening relationships with communities and other key stakeholders. Investigating questions around decision-making and stakeholder influences can help to uncover unique dynamics regarding the nature, form, and approach of professional sport CP and can elicit greater insight into this strategic practice.

Another limitation of our study is that it considered charitable giving as the dependent variable indicating the level of philanthropy. We did not take into account other forms of giving such as in-kind donations or other types of charitable investments made directly by a team that may also signal a team's social impact efforts. These forms of charitable behavior are also part of the broader portfolio of practices around social impact and choices around these efforts can be influenced by organizational leaders. Lastly, our research is limited to the US context. Professional sport teams in other countries may face unique institutional, regulative, and social contexts; there are also distinct governance and ownership structures of professional sport teams around the world. Thus, it would be interesting for future research to explore how the influence of team owners on CP differs in various cultural and geographical contexts.

## Data Availability Statement

The raw data supporting the conclusions of this article will be made available by the authors, without undue reservation.

## Author Contributions

KB drafted the introduction, literature review, and discussion. DY advised data cleaning and data analysis and drafted methods and results. All authors were part of the research from the beginning and contributed to study design, hypotheses development, data collection, writing manuscript. All authors contributed to the article and approved the submitted version.

## Conflict of Interest

The authors declare that the research was conducted in the absence of any commercial or financial relationships that could be construed as a potential conflict of interest.

## Publisher's Note

All claims expressed in this article are solely those of the authors and do not necessarily represent those of their affiliated organizations, or those of the publisher, the editors and the reviewers. Any product that may be evaluated in this article, or claim that may be made by its manufacturer, is not guaranteed or endorsed by the publisher.
